# Hospital admissions in children who had pelvicalyceal diltation detected on ultrasound scan during pregnancy: an e-cohort study

**DOI:** 10.23889/ijpds.v1i1.326

**Published:** 2017-04-19

**Authors:** Melissa Wright, Shantini Paranjothy, David Fone, Sinead Brophy, Joanne Demmler

**Affiliations:** 1 Cardiff University; 2 Swansea Univeristy

## Objective

To explore whether children with pelvicalyceal dilatation (PCD, a marker detected during the 18-20 week gestation ultrasound scan in which there is enlargement of tubes that collect urine in the kidney) have more hospital admissions for kidney problems in childhood compared to children without the marker. 

## Approach

We were funded by NISCHR to study outcomes associated with markers of uncertain significance at the second trimester anomaly scan (Welsh Study of Mothers and Babies). Data collected in the WSMB was uploaded to the Secure Anonymised Information Linkage (SAIL) databank and record linked to hospital activity data. Patterns of hospital admissions for renal causes were described and compared between those with no markers and those with PCD. Children were followed up from birth until 31st December 2014 or until the age of 5. A Cox Proportional Hazard Model was used to investigate the impact of PCD on time to first presentation.

## Results

Of the WSMB cohort, 20,834 children were eligible for inclusion in analyses. Those with PCD had 6.29 times the hazard of a renal admission compared to those without the marker (95% CI: 3.69 to 10.72). Children with PCD were more likely to have multiple renal admissions to hospital - median (interquartile range) number of renal admissions, 2.5 (1 to 5) compared to 1 (1 , 1) in children without markers. 

## Conclusion

Preliminary analysis suggests there is increased childhood renal morbidity associated with the presence of a PCD marker detected on the 18-20 week gestation ultrasound scan. These findings will inform the discussions clinicians have with parents when discussing the implications of this marker for the health of the chid.

